# Long-term outcomes of untreated cerebral cavernous malformations: a prospective, population-based cohort study

**DOI:** 10.1016/j.lanepe.2025.101410

**Published:** 2025-09-01

**Authors:** Abel Clemens Adriaan Sandmann, William Peter Vandertop, Philip Michael White, Dagmar Verbaan, Jonathan M. Coutinho, Rustam Al-Shahi Salman, Scottish Audit of Intracranial Vascular Malformations (SAIVMs) Steering Committee, Carl E. Counsell, Hazel Dodds, Gillian E. Stewart, Edward J. St George, Vakis Papanastassiou, Charles P. Warlow, Vaughn Ritchie, Richard C. Roberts, Robin J. Sellar, Jo J. Bhattacharya

**Affiliations:** a Amsterdam UMC, Department of Neurology, University of Amsterdam, Amsterdam, the Netherlands; b Amsterdam Neuroscience, Neurovascular Disorders, Amsterdam, the Netherlands; c Amsterdam UMC, Department of Neurosurgery, University of Amsterdam, Amsterdam, the Netherlands; d Translational and Clinical Research Institute, Newcastle University, Newcastle upon Tyne, United Kingdom; e Centre for Clinical Brain Sciences, University of Edinburgh, Edinburgh, United Kingdom

**Keywords:** Cavernous malformation, Clinical course, Natural history, Prognosis, Treatment, Conservative management, Surgery

## Abstract

**Background:**

Treatment decisions for cerebral cavernous malformations (CCM) currently rely on risk extrapolation from short-term studies, despite potential variations over time. This study aims to quantify the risks and functional outcomes of untreated CCMs during long-term follow-up.

**Methods:**

This population-based study included people aged ≥16 years in Scotland who were newly diagnosed with CCM between 1 January 1999 and 31 December 2003 or 1 January 2006 and 31 December 2010, using brain MRI or pathology. We analysed clinical events and functional outcomes using the Oxford Handicap Scale (OHS) during prospective follow-up without CCM intervention until 31 December 2023. The primary outcome was a composite of symptomatic intracranial haemorrhage (ICH) or new, non-haemorrhagic, persistent/progressive focal neurological deficit (FND) definitely or possibly related to CCM (ICH/FND).

**Findings:**

Among 300 patients (median age 44 years [IQR 32–57], 159 [53%] female, 48 [16%] brainstem CCM) included during 1999–2003 or 2006–2010, 81 (27%) presented with ICH/FND, 88 (29%) with epileptic seizure(s), and 131 (44%) incidentally. Over 4779 person-years of follow-up (completeness 95%), 44 patients were censored after microsurgical resection (n = 41) and stereotactic radiosurgery (n = 3). During a median untreated follow-up of 15 years (IQR 8–20), 40 (13%) patients experienced ICH/FND, 72 (24%) experienced dependence (OHS score 3–5), and 53 (18%) died (n = 7 [2%] related to CCM). The hazard of recurrent ICH/FND was higher than the first-event hazard (hazard ratio 8.66 [95% CI 4.44–16.90], p < 0.0001). The hazard rate of recurrent ICH/FND declined approximately 50-fold from 0.109 (95% CI 0.074–0.160) over the first five years to 0.002 (0.000–0.015) in the subsequent 20 years (p < 0.0001).

**Interpretation:**

The risk of a first ICH/FND from CCM is low, while the risk of recurrence is significantly higher, although the recurrence risk declines dramatically five years after a first ICH/FND. These long-term findings can guide clinical decision-making, and suggest focussing on a 5-year risk horizon rather than extrapolating annual risks to patients' lifetimes.

**Funding:**

10.13039/501100000265Medical Research Council, 10.13039/100014589Chief Scientist Office of the Scottish Government, and 10.13039/501100000364Stroke Association.

**Research in context**

**Evidence before this study**

We performed a systematic search (Appendix 11) for research articles indexed in OVID Medline and Embase from inception to 1 April 2025, that described original studies of ≥20 adult patients with cerebral cavernous malformations (CCM), who were followed during a quantified period of follow-up and in whom clinical events were analysed. We used the literature search of the individual patient data meta-analysis on the untreated clinical course of CCM, and combined the search terms with terms for “prognosis”, “natural history”, and “cohort study”. We identified 47 original studies (Appendix 12), all but one of which were hospital-based. The only population-based study was the initial cohort of patients newly diagnosed with CCM in Scotland, who were also included in the current study. Ten studies were prospective and 37 were (partly) retrospective. Six studies without selection criteria for CCM diagnosis, location, or multiplicity had a mean follow-up of >5 years (range 5.2–10.0), in which the pooled rate of a first CCM-related haemorrhage during follow-up was 3.2% per person-year.

**Added value of this study**

To the best of our knowledge, this study of CCM prognosis incorporates the longest average follow-up duration in the current literature. Using a population-based study design, restricted to incident cases of CCM, we assessed the untreated clinical course of CCMs during prospective follow-up (completeness 95%, median 15 years). We found an annual rate of first intracranial haemorrhage (ICH) during follow-up of 1.3% per person-year during the 0–5 year epoch, 0.3% per person-year during the 5–10 year epoch, and 0.1% during the >10 year epoch, which indicates a more favourable long-term prognosis of CCMs than previously thought. We further show that the risk of a first ICH (or focal neurological deficit [FND]) is low and remains constant over time, yet the risk of recurrent ICH/FND was highest after diagnosis, but declines during follow-up. While current best estimates are available over five years after diagnosis, we show that the risk of recurrent ICH/FND is 50-fold lower beyond five years of follow-up. Moreover, long-term functional outcomes improve over time, with three-quarters remaining functionally independent during follow-up, and death related to CCM is very rare in the long term.

**Implications of all the available evidence**

Long-term clinical and functional outcomes of patients with untreated CCMs improve over time. Risks of conservative management change during follow-up, indicating that extrapolation of annual risks to patients' lifetimes is inappropriate. Clinical practice should focus on a 5-year time horizon to which the up-front risks of CCM interventions are compared, as thereafter CCMs often become quiescent.

## Introduction

Cerebral cavernous malformations (CCM) are common intracranial vascular malformations, occurring at an estimated prevalence of 0.16–0.46%,^1^^,^^2^ and an incidence of approximately 0.5 people per 100,000 per year in population-based studies.^3^^,^^4^ The majority of CCMs occur sporadically, whilst approximately 20% of cases are familial, characterised by multiple CCMs,^5^ and they can also appear de novo after radiation exposure.^6^ Diagnosis of CCM often occurs incidentally, but CCMs can also cause intracranial haemorrhage (ICH), new non-haemorrhagic focal neurological deficit (FND), and epileptic seizures.^7^ Since CCMs are often diagnosed in young adults,^8^ they pose long-term cumulative risks that may lead to impaired quality of life, primarily due to reduced mental health.^9^

The treatment of patients with CCM frequently poses challenging decisions. CCM interventions (i.e., microsurgical resection or stereotactic radiosurgery) are used for prevention of future symptoms but carry a risk of disabling or fatal complications.^10^^,^^11^ A pilot phase randomised trial has not demonstrated a difference between CCM intervention and medical management alone, but it showed that a main phase was feasible.^12^ Until a definitive randomised controlled trial is performed, clinicians and patients must compare the up-front risks of CCM intervention indirectly to the risks of medical management alone, particularly the risk of (recurrent) symptomatic ICH and long-term disability.^13^

In clinical practice, the cumulative risks of conservative management are estimated by extrapolating annual risks reported by short-term, mostly retrospective, hospital-based cohort studies to a patient's lifetime. This approach favours intervention when life expectancy is longer and the estimated up-front risk of intervention is lower. However, this may not be valid if untreated risks change over time. Long-term cohort studies are needed to better understand the outcome for people with CCM who are managed medically without intervention. Therefore, our objective was to analyse clinical and functional outcomes, and assess whether risks vary over time among patients with untreated CCMs during very long-term follow-up in a prospective, population-based cohort study.

## Methods

### Patient selection

In this prospective, population-based cohort study, patients with CCM were identified by the Scottish Audit of Intracranial Vascular Malformations (SAIVMs), details of which have been reported previously.^4^^,^^14^ Briefly, SAIVMs is a National Health Service clinical audit of adults who were aged 16 years or older and resident in Scotland when first diagnosed using brain MRI or pathology with any type of intracranial vascular malformation between 1 January 1999 and 31 December 2003 or 1 January 2006 and 31 December 2010. Patients were identified through multiple overlapping sources of case ascertainment, which included a Scotland-wide collaborative network of neurologists, neurosurgeons, stroke physicians, radiologists, and pathologists, and central registers of hospital discharge records and death certificates.^4^

The observational studies (involving collection and analysis of routinely recorded clinical data without any change to care and to which opt-out consent applied) and annual postal questionnaire studies (which involved additional self-reported data and required written opt-in informed consent at enrolment) were approved by the Multicentre Research Ethics Committee for Scotland (MREC/98/0/48) and Fife and Forth Valley Research Ethics Committee (08/S0501/76). The audit and research protocols were published on the SAIVMs website and with the Directory of Clinical Databases (DoCDat). This article adheres to the STROBE guidelines for observational studies.^15^

### Data collection

Baseline characteristics were collected from the hospital medical records and included demographics, medical history, medicines, and functional status at the initial presentation on the Oxford Handicap Scale (OHS), which is a variant of the modified Rankin Scale (mRS).^16^ Baseline was defined as the patient's presentation, determined by the date of symptom onset or medical consultation (if asymptomatic), from which there was prospective follow-up leading to a diagnosis of CCM. Prospective functional outcomes and clinical events during follow-up until 31 December 2023 were identified using annual surveillance of general practitioners and hospital medical records, as well as annual postal questionnaires on each anniversary of the CCM diagnosis to general practitioners and consenting participants with CCM.

Each CCM diagnosis was verified on the clinical diagnostic brain images by certified neuroradiologists according to established criteria.^17^ Additionally, data on coexistent intracranial vascular malformations, CCM location, and CCM size were collected. Location was categorised as supratentorial, infratentorial, or supra- and infratentorial, with the first two applicable to single or multiple CCMs, and the latter only to multiple CCMs. Additionally, we noted the presence of a brainstem CCM separately to capture all patients who harboured a CCM in this location. For CCM size, we measured the largest diameter in any direction. In the case of multiple CCMs, we assessed the symptomatic CCM, or the largest CCM if the patient was asymptomatic.

### Event classification

The initial mode of presentation was classified using brain images and pathological reports. An initial presentation was incidental if the patient had been asymptomatic or if their symptoms (e.g., headache) could not be related to the CCM. Clinical events of symptomatic ICH at the initial presentation or during follow-up were distinguished from new FND based on the presence or absence of timely radiological, pathological, surgical, or cerebrospinal fluid evidence of recent haemorrhage.^7^ Events were classified as epileptic seizures if there was no radiological evidence that the seizures were provoked by a concomitant ICH. Events of FND were classified as transient (symptoms lasting <24 h), persistent (≥24 h), or progressive (≥24 h and intensifying).

Clinical events were classified as definitely, possibly, or not attributable to CCM. An event was possibly related to CCM if the clinical features were anatomically referable to the CCM, but another cause (e.g., ischaemic stroke) was possible, and brain imaging neither identified CCM haemorrhage nor confirmed an alternative cause. Outcome events during follow-up were assessed using the clinical, radiological, and pathological information available. For each outcome event, raw imaging data were retrieved, and on the rare occasion that these were unavailable, we used the radiological report. The cause of a death was determined by reviewing death certificates, autopsy reports if post-mortem examination had been done, and hospital medical records and clinical brain images if the death had occurred during a hospital admission.

### Outcomes of interest

The primary outcome was a composite of symptomatic ICH or new persistent/progressive non-haemorrhagic FND (ICH/FND) definitely or possibly related to the CCM.^14^ These events were combined in a composite outcome, as their severities seem similar,^18^ and some new FND might be undetected haemorrhages.^7^ New non-haemorrhagic FND included events of FND with timely brain imaging using the appropriate modality ruling out recent haemorrhage, or cases where imaging was performed with an inappropriate modality or too late to assess the presence of recent haemorrhage.^7^ We analysed persistent and progressive FND, but not transient FND lasting <24 h, as the latter does not affect clinical decision-making because of the lack of long-term effects.

In addition to a composite outcome, we also quantified symptomatic ICH alone to facilitate comparison with studies that focused primarily on ICH. Other outcomes of interest included epileptic seizure(s) definitely or possibly related to CCM, dependence (OHS score 3–5 during a prospective annual follow-up assessment), and death related to CCM.

### Statistical analysis

We quantified the completeness of follow-up data accrued as a proportion of the total potential follow-up that could have been obtained either before death or until 31 December 2023, which marked the end of follow-up in SAIVMs.^19^ We censored follow-up at the earliest occurrence of any of the following: first CCM intervention, death, or last available follow-up. We analysed functional outcomes on the OHS at each anniversary of CCM diagnosis during untreated follow-up using per-year proportions, in which patients who died were not directly censored, but remained classified as dead until 2023.

We performed several time-to-event analyses after any presentation using Kaplan–Meier estimates and Cox proportional hazards regression models. We used a minimum of eight events per covariate to determine the number of covariates in the model.^20^ We sequentially added the following six covariates to the model: age, sex, presentation with ICH/FND (versus the remaining modes of presentation), presence (versus absence) of a brainstem CCM, largest CCM diameter, and multiple (versus single) CCM. These covariates were selected based on the importance of demographic adjustment in epidemiological research, clinical relevance to CCM, and known or hypothesised risk factors for the primary outcome.^14^^,^^21^ We used log-log curves to assess whether proportional hazards assumptions were fulfilled, and if a covariate violated this assumption, we replaced it according to the prespecified sequence. We primarily analysed events that were definitely or possibly related to CCM, and performed sensitivity analysis to assess whether restricting to events definitely related to CCM affected the results.

In addition to Kaplan–Meier analysis of the primary outcome after any presentation, we analysed first and recurrent ICH/FND. First ICH/FND was quantified among patients who presented incidentally or with epileptic seizure(s), which started at presentation and stopped at the first ICH/FND during follow-up or date of censoring, whichever occurred first. We analysed recurrent ICH/FND among patients who initially presented with this event or had their first event during follow-up (after a presentation with epileptic seizure(s) or incidentally). The latter analysis started at the first ICH/FND (at presentation or during follow-up) and ended at the earliest occurrence of either a subsequent event or censoring. We also performed sensitivity analysis restricted to events of ICH/FND that were definitely related to CCM. Additionally, we analysed first and recurrent events of symptomatic ICH alone: the first event among patients who presented incidentally, with epileptic seizure(s), or with FND, and recurrence among those who had presented with symptomatic ICH or experienced their first event during follow-up.

We compared the Kaplan–Meier estimates of first and recurrent ICH/FND or ICH alone using long-rank tests and hazard ratios (HR). To assess whether the risk of the primary outcome of ICH/FND varied over time, we calculated hazard rates for specific time periods if there were sufficient events: annually for each of the first five years of follow-up, for these five years combined, and for the subsequent years of follow-up. We used a likelihood ratio test to test the added value of differentiating hazard rates between the first five years and the years thereafter (with respect to assuming a constant hazard over time). To model the relationship between hazard rates over time, we used a smoothing spline to estimate the hazard function. Analyses were done using IBM SPSS Statistics version 28,^22^ MedCalc Statistical Software version 22,^23^ and RStudio version 2022.2.3.^24^

### Funding and responsibility

The study sponsors had no role in the study design, the collection, analysis, and interpretation of data, the writing of the report, or the decision to submit the paper for publication.

## Results

### Baseline and follow-up

From 1 January 1999 to 31 December 2003 and 1 January 2006 to 31 December 2010, 306 adult residents in Scotland were newly diagnosed with one or more definite CCM (295 on brain MRI, six at autopsy and five after surgical excision). After omitting six patients whose CCM were first incidentally diagnosed at autopsy and who did not contribute to the outcome analyses, we included 300 patients. The median age at the initial presentation was 44 years (IQR 32–57) and 159 (53%) patients were women (Table 1). Fifty (17%) patients initially presented with symptomatic ICH, and 31 (10%) with FND, in whom symptoms were transient (n = 7), persistent (n = 20), or progressive (n = 4). Three patients had an unrelated intracranial aneurysm, and two had an unrelated arteriovenous malformation. Multiple CCMs occurred in 57 (19%) patients, and 48 (16%) had a brainstem CCM (baseline characteristics of these patients are provided in Appendix 1).

**Table 1:** Baseline characteristics.

	All patients (n = 300)	Incidental presentation (n = 131)	Presentation with ICH or FND (n = 81)	Presentation with epileptic seizure(s) unprovoked by ICH (n = 88)
Age (years)	44 (32–57)	48 (39–59)	42 (33–60)	36 (26–49)
Sex^a^				
Female	159 (53%)	76 (58%)	48 (59%)	35 (40%)
Male	141 (47%)	55 (42%)	33 (41%)	53 (60%)
Epileptic seizure(s) at presentation	100 (33%)	0 (0%)	12 (15%)	88 (100%)
Antithrombotic drugs	32 (11%)^b^	23 (18%)^b^	7 (9%)	2 (2%)
Antiplatelet agents	29 (10%)	21 (16%)	6 (7%)	2 (2%)
Anticoagulants	7 (2%)	6 (5%)	1 (1%)	0 (0%)
Multiple CCMs	57 (19%)	18 (14%)	17 (21%)	22 (25%)
Location of CCM				
Supratentorial	215 (72%)	100 (76%)	37 (46%)	78 (89%)
Infratentorial	54 (18%)	21 (16%)	33 (41%)	0 (0%)
Supra- and infratentorial	31 (10%)	10 (8%)	11 (14%)	10 (11%)
Brainstem CCM	48 (16%)	12 (9%)	30 (37%)	6 (7%)
Largest CCM diameter (mm)	12 (8–18)	10 (7–13)	15 (10–20)	12 (10–18)
Associated DVA	28 (9%)	16 (12%)	9 (11%)	3 (3%)
OHS score at presentation				
0	35 (12%)	35 (27%)	0 (0%)	0 (0%)
1	97 (32%)	55 (42%)	24 (30%)	18 (20%)
2	141 (47%)	30 (23%)	42 (52%)	69 (78%)
3	19 (6%)	6 (5%)	12 (15%)	1 (1%)
4	7 (2%)	4 (3%)	3 (4%)	0 (0%)
5	1 (0%)	1 (1%)	0 (0%)	0 (0%)

Data are median (IQR) or number (%); data on ethnicity were not available for all patients in SAIVMs, as they were not routinely collected.

CCM, cerebral cavernous malformation; DVA, developmental venous anomaly; FND, focal neurological deficit; ICH, intracranial haemorrhage; IQR, interquartile range; OHS, Oxford Handicap Scale.

a Sex assigned at birth.

b Four patients used both antiplatelet agents and anticoagulants.

Patients were followed for 4779 person-years (median 16 years, IQR 13–21, range 0–25) out of 5017 potential person-years, resulting in an overall completeness of 95%. Subsequently, follow-up was censored due to CCM intervention for 44 (15%) patients (n = 41 surgical excision, n = 3 stereotactic radiosurgery; in 26 of whom intervention occurred in the first year following the initial presentation). CCM intervention was performed in 36/169 (21%) patients who presented with symptoms (19/81 [23%] who presented with ICH/FND) and 3/48 (6%) patients with a brainstem CCM. The median untreated follow-up was 15 years (IQR 8–20, range 0–25, 4135 person-years), and 220 (73%) patients had more than 10 years of follow-up.

### Clinical events during follow-up

**Fig. 1:**
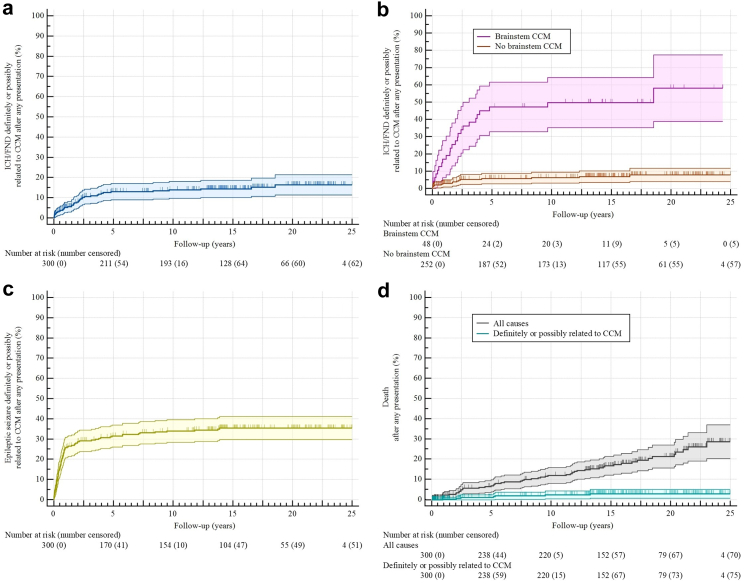
**Kaplan–Meier analyses for the progression to a first outcome event during follow-up after any presentation; a: ICH/FND definitely or possibly related to CCM (blue); b: ICH/FND definitely or possibly related to CCM stratified by presence (purple) or absence (orange) of brainstem CCM, the risk was higher for patients with brainstem CCM than those without (HR 9.42 [95% CI 4.99–17.78], p < 0.0001); c: epileptic seizure definitely or possibly related to CCM (yellow); d: death due to all causes (grey), and death definitely or possibly related to CCM (cyan). CCM, cerebral cavernous malformation; ICH/FND, symptomatic intracranial haemorrhage or new persistent/progressive focal neurological deficit**.

Overall, 40 (13%) patients experienced the primary outcome of ICH/FND definitely or possibly related to CCM during follow-up (Table 2 and Fig. 1a). The risk of ICH/FND was higher in association with presentation with ICH/FND and brainstem CCM (adjusted HR 5.52 [95% CI 2.54–11.99], p < 0.0001, and adjusted HR 4.62 [95% CI 2.24–9.52], p < 0.0001, Appendix 2, and p < 0.0001, log-rank test, Fig. 1b). Presentation with ICH/FND and brainstem CCM remained significantly associated with an increased risk in sensitivity analysis of ICH/FND that were only definitely related to CCM (Appendix 3), as well as in the analysis of symptomatic ICH alone (Appendix 4). Epileptic seizure(s) during follow-up were experienced by 98 (33%) patients (Fig. 1c).

**Table 2:** Outcomes during follow-up.

	Definitely or possibly related to CCM	Definitely related to CCM
Symptomatic outcome event^a^		
ICH/FND	40 (13%)^c^	30 (10%)^d^
Symptomatic ICH	21 (7%)	19 (6%)
New persistent/progressive FND	24 (8%)	15 (5%)
Epileptic seizure	98 (33%)	89 (30%)
Cumulative 5-year risk		
ICH/FND	13% (9%–17%)	11% (7%–14%)
Brainstem CCM	47% (33%–62%)	41% (26%–55%)
No brainstem CCM	6% (3%–9%)	4% (2%–7%)
Symptomatic ICH	6% (3%–9%)	6% (3%–9%)
Epileptic seizure	32% (26%–37%)	29% (23%–34%)
Cumulative 10-year risk		
ICH/FND	14% (10%–18%)	11% (7%–15%)
Brainstem CCM	50% (35%–64%)	43% (29%–57%)
No brainstem CCM	6% (3%–9%)	4% (2%–7%)
Symptomatic ICH	8% (4%–11%)	7% (4%–10%)
Epileptic seizure	34% (28%–40%)	31% (25%–36%)

	All causes	Definitely or possibly related to CCM	Definitely related to CCM
Death^b^	53 (18%)	7 (2%)	5 (2%)
Cumulative 5-year risk	7% (4%–10%)	1% (0%–3%)	1% (0%–2%)
Cumulative 10-year risk	12% (8%–16%)	2% (0%–4%)	2% (0%–3%)

Data are number (%) or risk (95% CI).

CCM, cerebral cavernous malformation; CI, confidence interval; FND, focal neurological deficit; ICH, intracranial haemorrhage; ICH/FND, symptomatic ICH or new persistent/progressive FND.

a Before first CCM intervention, death, or end of follow-up, whichever occurred first.

b Before first CCM intervention or end of follow-up, whichever occurred first.

c Five patients experienced symptomatic ICH as well as new persistent/progressive FND definitely or possibly related to CCM during follow-up.

d Four patients experienced symptomatic ICH as well as new persistent/progressive FND definitely related to CCM during follow-up.

### Death and functional outcomes

For 53 (18%) patients, follow-up ended because they died (Fig. 1d). Death was related to CCM in 7 (2%) patients (n = 5 definitely related to CCM, n = 2 possibly related to CCM; details are provided in Appendix 5).

**Fig. 2:**
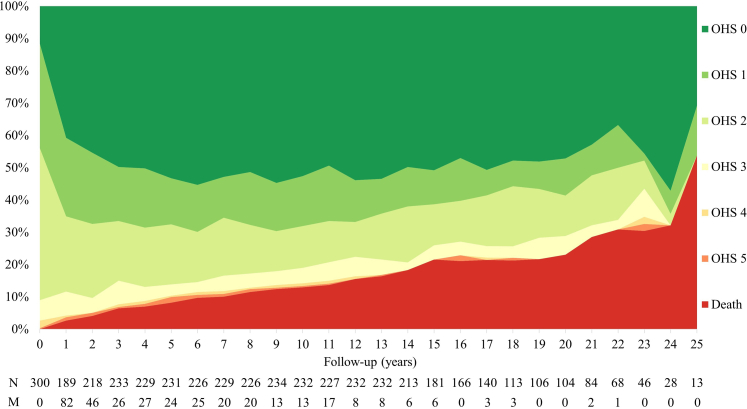
**Distribution of the Oxford Handicap Scale (OHS) scores at the initial presentation and each anniversary of CCM diagnosis during prospective annual follow-up; censoring occurred at intervention or last available follow-up; patients who died were not directly censored, but remained classified as dead until the last potential year of follow-up. CCM, cerebral cavernous malformation; M, missing; N, number included**.

Fig. 2 shows the distribution of OHS scores during annual follow-up for functional outcome at each anniversary of CCM diagnosis. The time between the date of the initial presentation and the date of the notification to SAIVMs was ≥1 year for 45 (15%) patients and ≥2 years for 19 (6%) patients (median 4 months [IQR 1–9]), which partly explains the relatively high number of missing assessments at year 1 and 2. Seventy-two (24%) patients experienced dependence (OHS score 3–5) at least once. The risk of dependence was lower in women (adjusted HR 0.54 [95% CI 0.33–0.87], p = 0.011, Appendix 6).

### First and recurrent ICH/FND

**Fig. 3:**
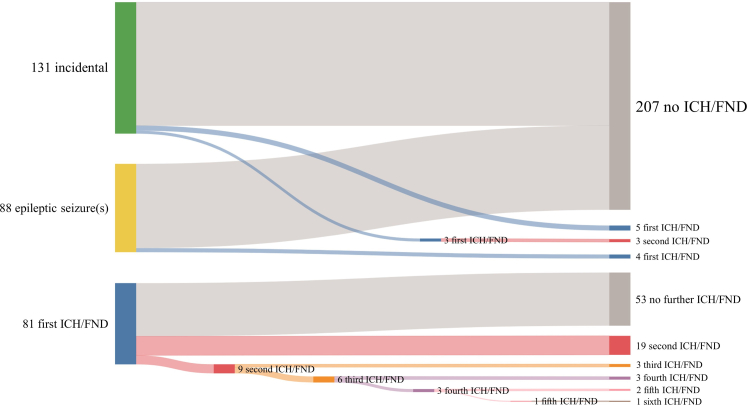
**Sankey diagram for events of ICH/FND definitely or possibly related to CCM; the nodes on the left represent the initial presentation; the grey flows are the patients who did not experience (further) ICH/FND during follow-up, and the coloured flows the patients who experienced ICH/FND during follow-up; the nodes on the right show the total number of events of ICH/FND experienced at the initial presentation and during follow-up. CCM, cerebral cavernous malformation; ICH/FND, symptomatic intracranial haemorrhage or new persistent/progressive focal neurological deficit**.

**Fig. 4:**
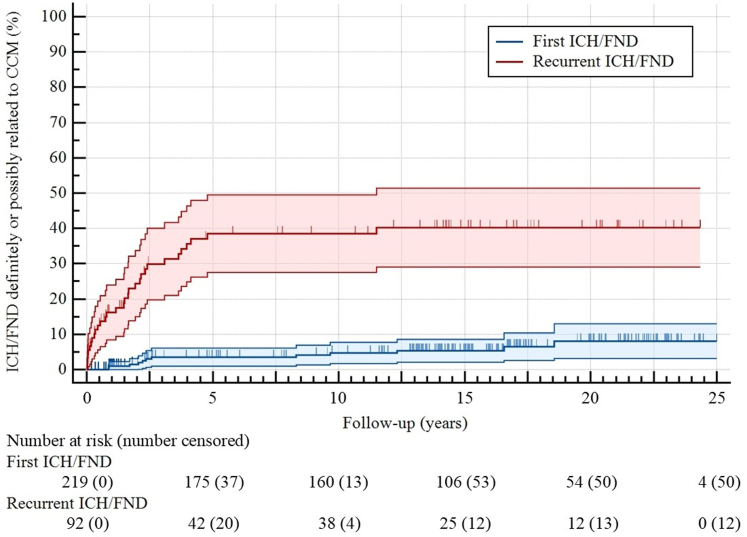
**Kaplan–Meier analysis for the progression to a first or recurrent ICH/FND definitely or possibly related to CCM; 5-year risk of a first ICH/FND: 4% (95% CI 1%–6%); 10-year risk of a first ICH/FND: 5% (2%–8%); 5-year and 10-year risks of recurrent ICH/FND: 39% (28%–49%); the risk of recurrent ICH/FND was higher than the risk of a first ICH/FND (HR 8.66 [95% CI 4.44–16.90], p < 0.0001). CCM, cerebral cavernous malformation; CI, confidence interval; HR, hazard ratio; ICH/FND, symptomatic intracranial haemorrhage or new persistent/progressive focal neurological deficit**.

Fig. 3 demonstrates the course of the primary outcome of ICH/FND definitely or possibly related to CCM at presentation and during follow-up. Of 219 patients who presented incidentally or with epileptic seizure(s), 12 patients experienced a first ICH/FND (10-year risk 5% [95% CI 2%–8%], Fig. 4), of whom one died due to a symptomatic ICH caused by a lobar CCM. Taking together the 11 survivors and the 81 patients who initially presented with ICH/FND, 31 experienced recurrent ICH/FND (10-year risk 39% [95% CI 28%–49%]), which was greater than the risk of a first ICH/FND (HR 8.66 [95% CI 4.44–16.90], p < 0.0001). Recurrent ICH/FND stratified by presence versus absence of brainstem CCM is shown in Appendix 7. In sensitivity analysis of events of ICH/FND that were only definitely related to CCM, the risk of recurrence remained greater than the risk of a first event (HR 23.64 [95% 8.26–67.66], p < 0.0001, Appendix 8).

**Fig. 5:**
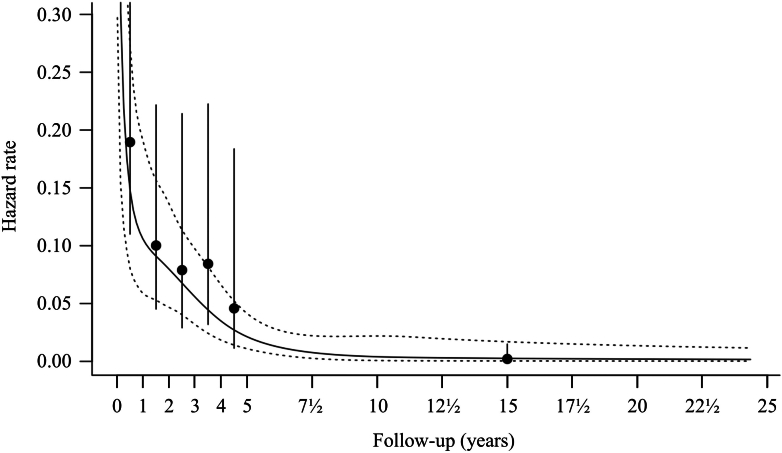
**Smoothing spline of the estimated hazard function of recurrent ICH/FND definitely or possibly related to CCM over time; the solid line represents the continuous hazard rate, and the dashed lines the 95% CI; the dots represent the annual hazard rates during the first five years and the hazard rate of the subsequent 20 years, and the vertical solid lines are the 95% CI of each interval; the hazard rate over the combined first five years decreased approximately 50-fold from 0.109 (95% CI 0.074–0.160) to 0.002 (0.000–0.015) over the 20 years thereafter (p < 0.0001, likelihood ratio test). CCM, cerebral cavernous malformation; CI, confidence interval; ICH/FND, symptomatic intracranial haemorrhage or new persistent/progressive focal neurological deficit**.

The number of first ICH/FND events was so small that determining whether first-event hazard rates varied over time was not feasible. The annual hazard rates (95% CI) of recurrent ICH/FND declined during the first five years of follow-up: 0.19 (0.11–0.33) in year 1, 0.10 (0.05–0.22) in year 2, 0.08 (0.03–0.21) in year 3, 0.08 (0.03–0.22) in year 4, and 0.05 (0.01–0.18) in year 5 (Fig. 5). The hazard rate of recurrent ICH/FND over the combined first five years of follow-up was 0.109 (0.074–0.160), after which the hazard rate decreased approximately 50-fold to 0.002 (0.000–0.015) during the subsequent 20 years (p < 0.0001, likelihood ratio test).

For symptomatic ICH alone (excluding events of new FND), of 250 patients who presented incidentally, with epileptic seizure(s), or with FND, 8 patients experienced a first ICH (n = 6 definitely related to CCM, n = 2 possibly related to CCM, Appendix 9). Of the 55 patients who experienced, at the presentation or during follow-up, a non-fatal first ICH definitely related to CCM, 16 experienced a recurrent event. The risk of recurrent ICH was greater than the risk of a first event (HR 17.16 [95% CI 6.69–44.02], p < 0.0001, Appendix 10).

## Discussion

In this prospective, population-based cohort study of patients with CCM undergoing long-term untreated follow-up, we found that the risk of a first ICH/FND was low and appeared constant over time, yet the risk of recurrent ICH/FND was highest shortly after the first event, but declined over five years, and was 50-fold lower in the 20 years of follow-up thereafter. Presentation with ICH/FND and brainstem CCM location were risk factors for ICH/FND during follow-up. Only 2% of patients died related to CCM, and approximately a quarter experienced dependence during follow-up.

Symptomatic ICH and new FND are among the most feared complications of untreated CCMs, given their potential to occur recurrently, possibly leading to disability and, although rare, death.^13^ The risks of these events have largely been estimated from short-term studies, with current best estimates available over five years after diagnosis. As reported by an individual patient data meta-analysis, the estimated 5-year rate of ICH/FND is 23%–51% for patients with a brainstem CCM, and 4%–22% for those without (upper value for those who initially presented with ICH/FND and lower value for other presentations).^25^ Additionally, the risk of recurrent ICH or FND is known to decline annually over the first five years of follow-up, but there has been uncertainty about the rate thereafter. The current study shows that this rate is very low in absolute terms and substantially lower than during the first five years.

During long-term follow-up, most patients remained free of symptomatic events, with only one-seventh undergoing CCM intervention. The rate of intervention is notably lower than the 27%–48% reported in retrospective, hospital-based studies with a similar sample size.^26^^,^^27^ This difference may reflect practice variation between countries but could also be due to selection bias in those studies. The intervention rate was slightly higher than in a study of patients who were managed conservatively as primary treatment strategy (excluding 4% who were primarily surgically treated), in which only one out of 20 patients required CCM intervention during a median follow-up of five years.^21^ The current long-term evidence of a low CCM intervention rate may reassure clinicians and patients considering medical management without intervention, especially when CCM is an incidental discovery.

Our data confirm that the annual risk of recurrent ICH/FND declines during the first five years of follow-up and exceeds the first-event risk, as reported in the only population-based study previously published in the literature, which included patients newly diagnosed with CCM in Scotland during 1999–2003.^14^ However, that study had a small sample size and lacked long-term follow-up (median 5 years). For improved precision, we doubled the sample size and followed up patients in the very long term. This resulted in capturing a greater number of outcome events, which permitted multivariable analyses. We sequentially added six prespecified candidate covariates to the models, using a minimum of eight events per covariate,^20^ although more recent methods exist for selecting covariates and determining the maximum number in multivariable models.^28^ We confirmed that an initial presentation with ICH/FND and presence of a brainstem CCM were risk factors for ICH/FND during follow-up. This aligns with findings from previous studies,^21^^,^^25^ although our study includes patients and follow-up data that partially overlap with the 5-year dataset used in the individual patient data meta-analysis on the untreated clinical course of CCM.^25^

We analysed functional outcomes on the OHS over up to 25 years of follow-up, showing improvement of the OHS score distribution during the first years after the initial presentation. This may indicate that physical recovery occurs after an—often-symptomatic—initial presentation, as stipulated in a previous study.^9^ Functional independence was maintained by three-quarters of patients during follow-up, with an increased risk of dependence for men. This in contrast to the general population, in which women are more likely to experience disability while men have higher mortality risks.^29^ We were unable to assess whether this finding was related to CCM because functional outcomes are not disease-specific and may be influenced by comorbidities. However, it is possible that CCM-related epilepsy contributed to the observed functional outcome. Furthermore, we found that the majority of deaths were unrelated to CCM, as only 2% of the patients died related to CCM over a median follow-up of 15 years. This low CCM-related mortality rate aligns with previous studies.^10^^,^^11^^,^^21^

This study was designed to minimise several potential sources of bias. Its population-based design, restricted to incident cases of CCM, minimised the selection bias often encountered in hospital-based studies. To reduce information bias, we used prospective follow-up, which had an overall completeness of 95% over a period of 25 years.^30^ We minimised detection and misclassification biases by using widely used diagnostic criteria and outcome definitions.^7^ However, despite these strengths, we may have missed some outcome events as we did not rely on scheduled study visits but only analysed clinical events reported to the general practitioner or resulting in hospitalisation. Additionally, some patients may not have reported all new events, as their CCM diagnosis was made more than two decades ago. However, if so, such events may also be clinically less relevant and relying solely on the available clinical information similar to everyday clinical practice probably further enhances the generalisability of our results.

Several other limitations may be present in this study. First, rates of ICH may have been underestimated, as not every epileptic seizure is studied with contemporaneous brain imaging in clinical practice, even though symptomatic ICH may present with seizures alone. Second, censoring patients at intervention may have led to informative censoring, as CCM interventions are often performed in patients perceived to have a poor prognosis. CCM intervention was done after almost one-quarter of presentations with ICH/FND, though the proportion was lower than those reported in hospital-based studies. Third, in the analyses to first and recurrent events, patients who experienced their first event during follow-up contributed parts of their follow-up to both groups that were compared. Fourth, we were unable to include all prespecified covariates in the multivariable models due to a low number of outcome events. Fifth, OHS score assessments were not timed in relation to outcome events but were conducted annually. Therefore, score increases may have been missed if patients were censored before the next annual assessment. Sixth, the year-1 and year-2 OHS scores were missing relatively frequently as the time between initial presentation and notification to SAIVMs often exceeded a year and/or due to delays in obtaining informed consent. Seventh, because we did not separately analyse patients with familial CCM, who usually have multiple CCMs, the generalisability of the results to such patients is uncertain.

The results from this study enhance our understanding of CCM prognosis and treatment, particularly in the absence of randomised controlled trials. While intervention is not indicated for incidental CCM and is commonly considered for patients at risk of recurrent ICH/FND, most existing literature focuses on the risk of a first event after the initial presentation. Our findings show that the risk of recurrent ICH/FND declines during untreated follow-up and is 50-fold lower beyond five years, indicating that the prognosis of these patients improves over time. Death related to CCM is rare and three-quarters of patients do not suffer dependence. These data offer insights to optimise the management of patients with CCM, demonstrating that extrapolation of annual risks to patients' lifetimes is inappropriate. These long-term findings are reassuring for patients who escape recurrent ICH/FND over five years after a first ICH/FND, and focus future randomised controlled trials and everyday clinical practice on a 5-year time horizon to which the up-front risks of CCM interventions are compared, as thereafter CCMs often become quiescent.

## Contributors

The corresponding author had full access to all study data, and other authors were not precluded from accessing the study data and take responsibility for the decision to submit the manuscript. Abel Clemens Adriaan Sandmann and Rustam Al-Shahi Salman have directly accessed and verified the underlying data reported in the manuscript. Abel Clemens Adriaan Sandmann: conceptualisation, formal analysis, funding acquisition, investigation, methodology, project administration, visualisation, writing - original draft, writing - review & editing. William Peter Vandertop: writing - review & editing. Philip Michael White: writing - review & editing. Dagmar Verbaan: conceptualisation, methodology, writing - review & editing. Jonathan M. Coutinho: conceptualisation, methodology, writing - review & editing. Rustam Al-Shahi Salman: conceptualisation, data curation, funding acquisition, investigation, methodology, resources, software, supervision, validation, writing - review & editing.

## Data sharing statement

The audit and research protocols were published on the SAIVMs website and with the Directory of Clinical Databases (DoCDat). Deidentified individual participant data that underlie the results reported in this article will be made available for scientific purposes upon formal request and consequent approval of the proposal after publication. Reasonable requests should be sent to the corresponding author.

## Declaration of interests

None of the authors received payment or compensation from a pharmaceutical company or any other agency for writing this article. Abel Clemens Adriaan Sandmann received financial travel support from European Stroke Organisation, Cultuurfonds, Dr. Jan Meerwaldt Foundation, Amsterdam University Fund, Royal Netherlands Academy of Arts & Sciences, CONTRAST 2.0 Consortium, Foundation “De Drie Lichten” in the Netherlands, and Remmert Adriaan Laan-Fonds (all payments were made to his institution directly or indirectly). William Peter Vandertop declares no conflicts of interest. Philip Michael White received institutional educational grant support from Medtronic and Stryker. Dagmar Verbaan declares no conflicts of interest. Jonathan M. Coutinho received financial support from Bayer and AstraZeneca (all fees were paid to his employer), and is co-founder and shareholder of TrianecT. Rustam Al-Shahi Salman received financial support from the British Heart Foundation, Chief Scientist Office Health Improvement, Recursion Pharmaceuticals, European Stroke Organisation and Novo Nordisk (all fees were paid to The University of Edinburgh), and from the UK Clinical Research Collaboration (paid via The University of Edinburgh).

## Appendix A Supplementary data

Appendices 1–12
