# Supplementary material for: Long-term outcomes of untreated cerebral cavernous malformations: a prospective, population-based cohort study
Source: Lancet Reg Health Eur. 2025 Sep 1;57:101410. doi: 10.1016/j.lanepe.2025.101410 (PMC12541635; doi:10.1016/j.lanepe.2025.101410)
Supplement: Appendices 1–12 [file mmc1.docx]

**Supplementary material**

Supplement to: *Long-term outcomes of untreated cerebral cavernous malformations: a prospective, population-based cohort study*

**1. Table. Baseline characteristics of all patients and stratified by presence or absence of brainstem CCM**

| **Variable** | **All patients (n=300)** | **Brainstem CCM (n=48)** | **No brainstem CCM (n=252)** |
| --- | --- | --- | --- |
| Age (years) | 44 (32-57) | 47 (38-60) | 42 (32-57) |
| Sex (female) | 159 (53%) | 27 (56%) | 132 (52%) |
| Mode of presentation |  | | |
| Symptomatic ICH | 50 (17%) | 16 (33%) | 34 (13%) |
| With epileptic seizure(s) | 12 (4%) | 0 (0%) | 12 (5%) |
| Epileptic seizure(s) | 88 (29%) | 6 (13%) | 82 (33%) |
| FND | 31 (10%) | 14 (29%) | 17 (7%) |
| Incidental | 131 (44%) | 12 (25%) | 119 (47%) |
| Antithrombotic drugs | 32 (11%)^1^ | 3 (6%) | 29 (12%)^1^ |
| Antiplatelet agents | 29 (10%) | 3 (6%) | 26 (10%) |
| Anticoagulants | 7 (2%) | 0 (0%) | 7 (3%) |
| Multiple CCMs | 57 (19%) | 19 (40%) | 38 (15%) |
| Location |  | | |
| Supratentorial | 215 (72%) | 0 (0%) | 215 (85%) |
| Infratentorial | 54 (18%) | 29 (60%) | 25 (10%) |
| Supra- and infratentorial | 31 (10%) | 19 (40%) | 12 (5%) |
| Largest CCM diameter (mm) | 12 (8-18) | 13 (10-17) | 12 (8-18) |
| Associated DVA | 28 (9%) | 1 (2%) | 27 (11%) |
| OHS score at presentation |  | | |
| 0 | 35 (12%) | 4 (8%) | 31 (12%) |
| 1 | 97 (32%) | 15 (31%) | 82 (33%) |
| 2 | 141 (47%) | 22 (46%) | 119 (47%) |
| 3 | 19 (6%) | 6 (13%) | 13 (5%) |
| 4 | 7 (2%) | 1 (2%) | 6 (2%) |
| 5 | 1 (0%) | 0 (0%) | 1 (0%) |

Data are median (IQR) or number (%); CCM, cerebral cavernous malformation; DVA, developmental venous anomaly; FND, focal neurological deficit; ICH, intracranial haemorrhage; IQR, interquartile range; OHS, Oxford Handicap Scale.

^1^Four patients used both antiplatelet agents and anticoagulants

**2. Table.** **Cox proportional hazards regression model for associations with the occurrence of symptomatic ICH or new persistent/progressive FND definitely or possibly related to CCM during follow-up**

| **Covariate** | **All patients (n=300)** | **Unadjusted HR (95% CI)** | **P-value** | **Adjusted HR (95% CI)** | **P-value** |
| --- | --- | --- | --- | --- | --- |
| Female (versus male) sex | 159 (53%) | 1·53 (0·81-2·91) | 0·19 | 1·20 (0·60-2·39) | 0·60 |
| Presentation with (versus without) ICH/FND | 81 (27%) | 8·94 (4·53-17·61) | **<0·0001** | 5·52 (2·54-11·99) | **<0·0001** |
| Presence (versus absence) of brainstem CCM | 48 (16%) | 9·42 (4·99-17·78) | **<0·0001** | 4·62 (2·24-9·52) | **<0·0001** |
| Largest CCM diameter (1-mm increments) | 12 (8-18) | 1·00 (0·96-1·05) | 0·94 | 0·98 (0·92-1·04) | 0·52 |
| Multiple (versus single) CCM | 57 (19%) | 1·35 (0·64-2·83) | 0·43 | 1·23 (0·54-2·79) | 0·62 |

Data are number (%), median (IQR), or HR (95% CI); p-values in bold were considered statistically significant (p<0·05); CCM, cerebral cavernous malformation; CI, confidence interval; FND, focal neurological deficit; HR, hazard ratio; ICH, intracranial haemorrhage; ICH/FND, symptomatic ICH or new persistent/progressive FND; IQR, interquartile range; the variable age was not included because the proportional hazards assumption was violated.

**3. Table. Cox proportional hazards regression model for associations with the occurrence of symptomatic ICH or new persistent/progressive FND definitely related to CCM during follow-up**

| **Covariate** | **All patients (n=300)** | **Unadjusted HR (95% CI)** | **P-value** | **Adjusted HR (95% CI)** | **P-value** |
| --- | --- | --- | --- | --- | --- |
| Presentation with (versus without) ICH/FND | 81 (27%) | 23·76 (8·28-68·20) | **<0·0001** | 14·25 (4·72-43·05) | **<0·0001** |
| Presence (versus absence) of brainstem CCM | 48 (16%) | 11·64 (5·44-24·88) | **<0·0001** | 4·58 (2·08-10·10) | **<0·0001** |
| Multiple (versus single) CCM | 57 (19%) | 1·39 (0·60-3·24) | 0·45 | 1·30 (0·55-3·06) | 0·55 |

Data are number (%) or HR (95% CI); p-values in bold were considered statistically significant (p<0·05); CCM, cerebral cavernous malformation; CI, confidence interval; FND, focal neurological deficit; HR, hazard ratio; ICH, intracranial haemorrhage; ICH/FND, symptomatic ICH or new persistent/progressive FND; the variables age, sex, and largest CCM diameter were not included because the proportional hazards assumption was violated.

**4. Table. Cox proportional hazards regression models for associations with the occurrence of symptomatic ICH definitely related to CCM during follow-up**

| **Covariate** | **All patients (n=300)** | **Unadjusted HR (95% CI)** | **P-value** | **Adjusted HR (95% CI)** | **P-value** |
| --- | --- | --- | --- | --- | --- |
| **Model following the prespecified sequence of covariates** | | | | | |
| Female (versus male) sex | 159 (53%) | 1·22 (0·49-3·02) | 0·67 | 1·17 (0·47-2·92) | 0·73 |
| Presentation with (versus without) ICH/FND | 81 (27%) | 15·25 (5·77-40·27) | **<0·0001** | 15·21 (5·76-40·18) | **<0·0001** |
| **Model without the variable sex** | | | | | |
| Presentation with (versus without) sympt. ICH | 81 (27%) | 15·25 (5·77-40·27) | **<0·0001** | 9·99 (3·65-27·33) | **<0·0001** |
| Presence (versus absence) of brainstem CCM | 48 (16%) | 9·26 (3·65-23·52) | **<0·0001** | 5·29 (2·01-13·89) | **<0·0001** |

Data are number (%) or HR (95% CI); p-values in bold were considered statistically significant (p<0·05); CCM, cerebral cavernous malformation; CI, confidence interval; FND, focal neurological deficit; HR, hazard ratio; ICH, intracranial haemorrhage; ICH/FND, symptomatic ICH or new persistent/progressive FND; symptomatic ICH possibly related to CCM is not included in the table, but occurred once in two patients both during follow-up (after an incidental diagnosis and after a presentation with epileptic seizure(s)); the variable age was not included because the proportional hazards assumption was violated.

**5. Table. Causes of death for patients who died definitely or possibly related to CCM**

| **Age^1^** | **Sex** | **Mode of presentation** | **CCM location** | **Follow-up** | **Cause of death** | **Relation to CCM** |
| --- | --- | --- | --- | --- | --- | --- |
| 19 | M | Epileptic seizure(s) | Multiple supratentorial | 5 years | Found in bed after two epileptic seizures the day before, no cause identified at autopsy | Definite |
| 39 | M | Epileptic seizure(s) | Multiple supra- & infratentorial | 13 years | Refractory status epilepticus; septic shock after multiple organ failure | Definite |
| 48 | M | Incidental | Insula | 2 years | Post-mortem: symptomatic ICH from CCM and suffocation by foreign body | Definite |
| 72 | F | Focal neurological deficit | Brainstem | 1 year | Aspiration pneumonia most likely due to brainstem dysfunction due to CCM | Definite |
| 82 | F | Incidental | Parietal lobe | 10 years | Increasing frailty; aspiration pneumonia and sepsis after several epileptic seizures | Definite |
| 51 | M | Epileptic seizure(s) | Frontal lobe | 5 years | Post-mortem: likely coronary artery disease, but SUDEP possible | Possible |
| 58 | F | Incidental | Parietal lobe | 2 years | Post-mortem: aspiration pneumonia, COPD, no further details available | Possible |

CCM, cerebral cavernous malformation; COPD, chronic obstructive pulmonary disease; ICH, intracranial haemorrhage; SUDEP, sudden unexpected death in epilepsy.

^1^Age at the initial presentation

**6. Table. Cox proportional hazards regression model for associations with the occurrence of dependence (OHS score 3-5) during follow-up**

| **Covariate** | **All patients (n=300)** | **Unadjusted HR (95% CI)** | **P-value** | **Adjusted HR (95% CI)** | **P-value** |
| --- | --- | --- | --- | --- | --- |
| Age (10-year increments) | 44 (32-57) | 1·08 (0·93-1·25) | 0·35 | 1·09 (0·93-1·27) | 0·29 |
| Female (versus male) sex | 159 (53%) | 0·55 (0·34-0·88) | **0·012** | 0·54 (0·33-0·87) | **0·011** |
| Presentation with (versus without) ICH/FND | 81 (27%) | 0·85 (0·49-1·48) | 0·56 | 0·74 (0·41-1·36) | 0·34 |
| Presence (versus absence) of brainstem CCM | 48 (16%) | 1·38 (0·78-2·44) | 0·27 | 1·49 (0·78-2·85) | 0·23 |
| Largest CCM diameter (1-mm increments) | 12 (8-18) | 0·99 (0·96-1·03) | 0·69 | 1·01 (0·97-1·05) | 0·74 |
| Multiple (versus single) CCM | 57 (19%) | 1·12 (0·63-2·01) | 0·70 | 1·01 (0·54-1·90) | 0·97 |

Data are median (IQR), number (%), or HR (95% CI); p-values in bold were considered statistically significant (p<0·05); CCM, cerebral cavernous malformation; CI, confidence interval; HR, hazard ratio; ICH/FND, symptomatic intracranial haemorrhage or new persistent/progressive focal neurological deficit; IQR, interquartile range; OHS, Oxford Handicap Scale.

**7. Figure. Kaplan-Meier analysis for the progression to recurrent ICH/FND definitely or possibly related to CCM stratified by presence versus absence of brainstem CCM; CCM, cerebral cavernous malformation; ICH/FND, symptomatic intracranial haemorrhage or new persistent/progressive focal neurological deficit**


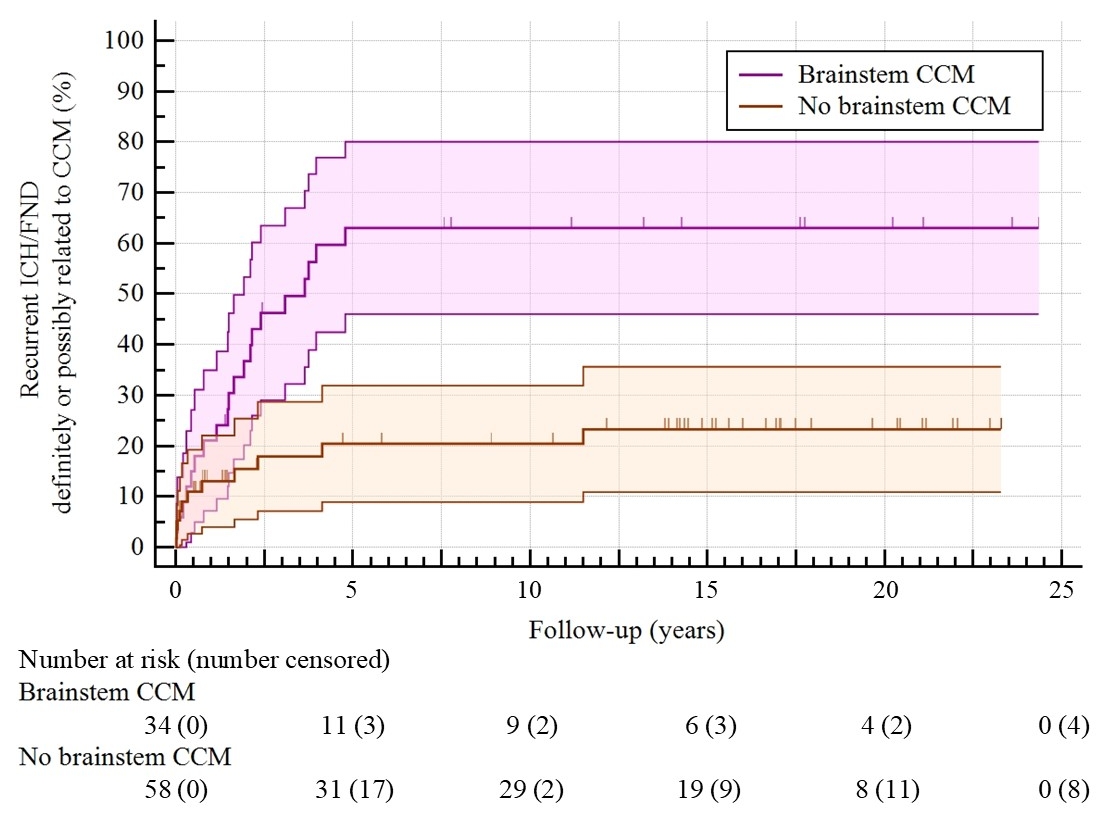


**8. Figure. Kaplan-Meier analysis for the progression to a first or recurrent ICH/FND definitely related to CCM; 5-year risk of a first ICH/FND: 2% (95% CI 0%-3%); 10-year risk of a first ICH/FND: 2% (0%-4%); 5-year and 10-year risks of recurrent ICH/FND: 37% (26%-49%); the risk of recurrent ICH/FND was higher than the risk of a first ICH/FND (HR 23·64, 95% 8·26-67·66, p<0·0001); CCM, cerebral cavernous malformation; CI, confidence interval; HR, hazard ratio; ICH/FND, symptomatic intracranial haemorrhage or new persistent/progressive focal neurological deficit**


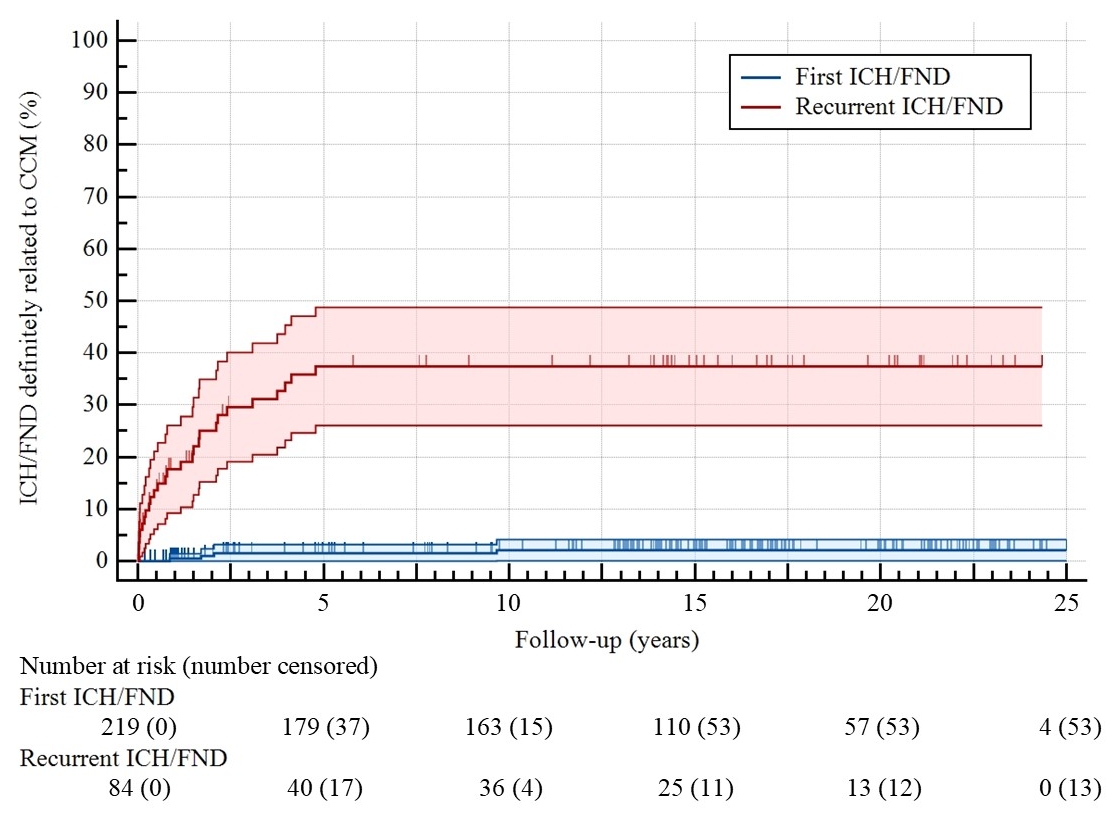


**9. Figure. Sankey diagram for events of symptomatic ICH definitely related to CCM (symptomatic ICH possibly related to CCM is not included in the figure, but occurred once in two patients both during follow-up; after an incidental diagnosis and after a presentation with epileptic seizure(s)); the nodes on the left represent the initial presentation; the grey flows are the patients who did not experience (further) symptomatic ICH during follow-up, and the coloured flows the patients who experienced symptomatic ICH during follow-up; the nodes on the right show the total number of events of symptomatic ICH experienced at the initial presentation and during follow-up; CCM, cerebral cavernous malformation; FND, focal neurological deficit; ICH, intracranial haemorrhage**


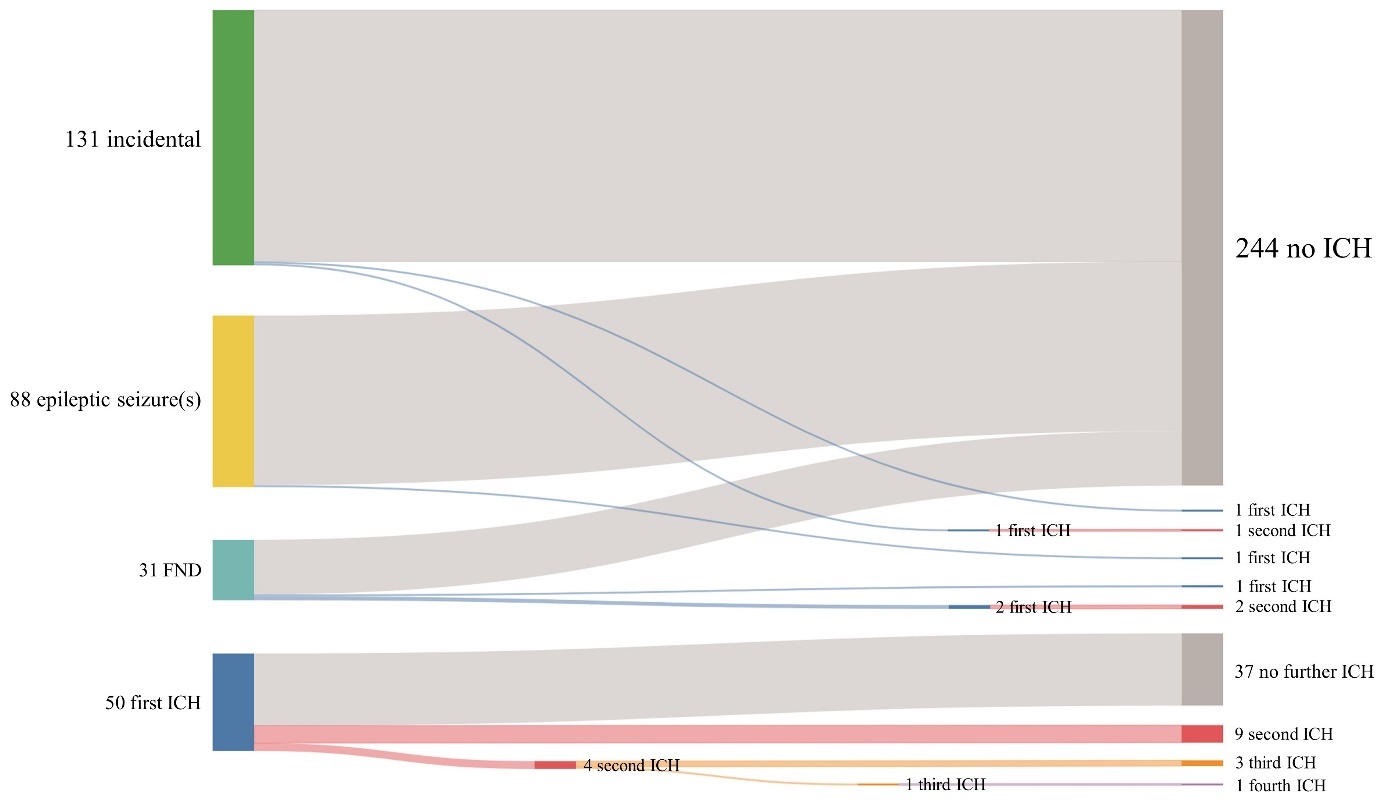


**10. Figure. Kaplan-Meier analysis for the progression to a first or recurrent symptomatic ICH definitely related to CCM (symptomatic ICH possibly related to CCM is not included in the figure, but occurred once in two patients both during follow-up; after an incidental diagnosis and after a presentation with epileptic seizure(s)); 5-year risk of first symptomatic ICH: 2% (95% CI 0%-4%); 10-year risk of first symptomatic ICH: 3% (1%-5%); 5-year risk of recurrent symptomatic ICH: 32% (18%-46%); 10-year risk of recurrent symptomatic ICH: 35% (21%-49%); the risk of recurrent symptomatic ICH was higher than the risk of a first symptomatic ICH (HR 17·16, 95% CI 6·69-44·02, p<0·0001); CCM, cerebral cavernous malformation; CI, confidence interval; HR, hazard ratio; ICH, intracranial haemorrhage**


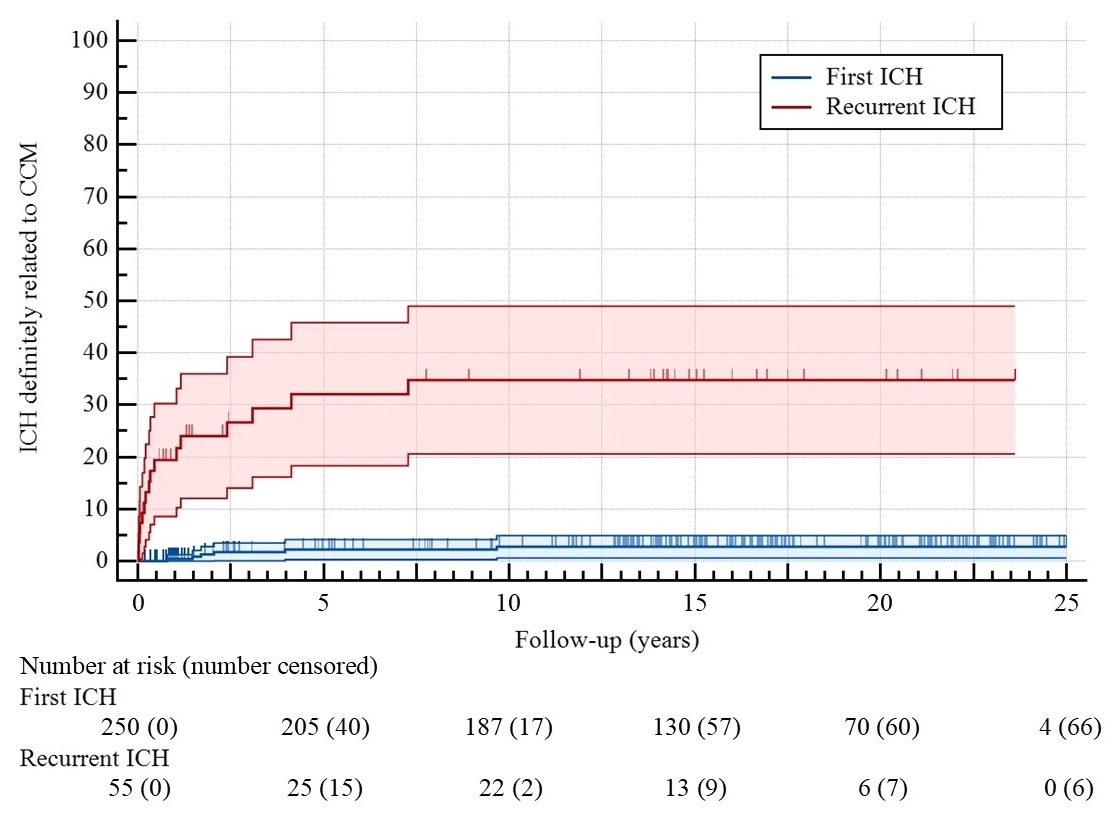


**11. Systematic search strategy**

**MEDLINE**

1. Hemangioma, Cavernous, Central Nervous System/

2. Hemangioma, Cavernous/

3. (cavernous adj5 (angioma* or hemangioma* or malformation*)).tw.

4. cavernoma*.tw.

5. 2 or 3 or 4

6. central nervous system/ or exp brain/ or exp cerebral arteries/

7. exp brain neoplasms/

8. (brain* or cerebral or intracerebral or central nervous system or intracranial or cerebellar or intraventricular or supratentorial).tw.

9. 6 or 7 or 8

10. 5 and 9

11. 1 or 10

12. Prognosis/ or treatment outcome/ or disease progression/ or natural history/ or cohort studies/ or mortality/ or survival analysis/ or survival rate/

13. (outcome* or prognosis or clinical course or natural history or disease progression or cohort stud* or prospective* or retrospective* or observation* or mortality or survival).tw.

14. 12 or 13

15. 11 and 14

**EMBASE**

1. Brain Hemangioma/

2. Cavernous Hemangioma/

3. (cavernous adj5 (angioma* or hemangioma* or malformation*)).tw.

4. cavernoma*.tw.

5. 2 or 3 or 4

6. central nervous system/ or exp brain/ or exp brain ventricle/ or exp brain artery/

7. exp brain tumor/

8. (brain* or cerebral or intracerebral or central nervous system or intracranial or cerebellar or intraventricular or supratentorial).tw.

9. 6 or 7 or 8

10. 5 and 9

11. 1 or 10

12. Prognosis/ or treatment outcome/ or disease progression/ or disease course/ or cohort analysis/ or mortality/ or survival analysis/ or survival rate/

13. (outcome* or prognosis or clinical course or natural history or disease progression or cohort stud* or prospective* or retrospective* or observation* or mortality or survival).tw.

14. 12 or 13

15. 11 and 14

**12. Table. Characteristics of studies of the untreated clinical course of at least 20 adult patients with cerebral cavernous malformation**

|  | Number of patients | Study design:  □ hospital-based  ■ population-based | Case identification:  □ retrospective  ■ prospective | Follow-up:  □ retrospective  ■ prospective | Follow-up duration (years) | | Other selection criteria |
| --- | --- | --- | --- | --- | --- | --- | --- |
|  |  |  |  |  | Mean | Max |  |
| Kim *et al.* 1997^1^ | 62 | □ | ? | □ | 1·9 | 4·0 | None |
| Ma *et al.* 2020^2^ | 282 | □ | ■ | □ | 1·9 | 3·5 | Bled at presentation |
| Labauge *et al.* 2001^3^ | 33 | ? | □ | ■ | 2·1 | 4·5 | Familial CCM and incidental |
| Carrion-Penagos *et al.* 2021^4^ | 192 | □ | ■ | ■ | 2·1 | ? | None |
| Robinson *et al.* 1991^5^ | 66 | □ | □ | □ | 2·2 | ? | None |
| Fritschi *et al.* 1994^6^ | 139 | □ | ? | □ | 2·2 | 11 | Brainstem CCM |
| Zabramski *et al.* 1994^7^ | 21 | □ | □ | ■ | 2·2 | 5·5 | Familial CCM |
| Gomez-Paz *et al.* 2020^8^ | 438 | □ | ■ | □ | 2·2^1^ | ? | None |
| Kalani *et al.* 2013^9^ | 64 | □ | □ | □ | 2·3 | ? | Pregnant women |
| Marques *et al.* 2023^10^ | 688 | □ | ■ | □ | 2·3 | ? | None |
| Ghannane *et al.* 2007^11^ | 79 | □ | ? | □ | 2·5 | 3·6 | None |
| Santos *et al.* 2022^12^ | 205 | □ | ? | □ | 2·6 | ? | Familial CCM |
| Santos *et al.* 2022^13^ | 238 | □ | ? | □ | 2·6 | ? | Multiple CCMs |
| Kondziolka *et al.* 1995^14^ | 122 | □ | ■ | □ & ■ | 2·8 | 6·8 | Conservative management |
| Porter *et al.* 1999^15^ | 100 | □ | □ | □ | 2·9 | 14 | Brainstem CCM |
| Jeon *et al.* 2014^16^ | 326 | □ | ■ | □ | 2·9 | ? | None |
| Bervini *et al.* 2019^17^ | 408 | □ | ■ | □ | 3·0 | ? | None |
| Santos *et al.* 2023^18^ | 42 | □ | □ | □ & ■ | 3·1 | ? | Bled twice |
| Chen *et al.* 2020^19^ | 731 | □ | ■ | □ & ■ | 3·2 | ? | None |
| Arauz *et al.* 2017^20^ | 99 | □ | □ | ■ | 3·3 | ? | Brainstem CCM, bled at present. |
| Zuurbier *et al.* 2023^21^ | 722 | □ | ■ | ■ | 3·3 | ? | Women |
| Al-Holou *et al.* 2012^22^ | 56 | □ | ■ | □ | 3·5 | ? | Age ≤25 years |
| Weinsheimer *et al.* 2023^23^ | 386 | □ | □ | ■ | 3·5 | 8·3 | Familial CCM |
| Dammann *et al.* 2016^24^ | 199 | □ | ■ | □ | 3·6 | 10·3 | Solitary sporadic CCM |
| Tian *et al.* 2017^25^ | 121 | □ | ■ | □ & ■ | 3·6 | ? | Thalamic CCM |
| Goldberg *et al.* 2018^26^ | 408 | □ | ■ | □ | 3·7 | ? | None |
| Porter *et al.* 1997^27^ | 110 | □ | ■ | □ & ■ | 3·8 | ? | None |
| Barker *et al.* 2001^28^ | 136 | □ | ■ | □ | 3·8 | ? | Bled at presentation |
| Schneble *et al.* 2012^29^ | 87 | □ | ■ | □ | 3·9 | ? | None |
| Kearns *et al.* 2019^30^ | 84 | □ | ■ | □ | 3·9 | ? | None |
| Hasegawa *et al.* 2002^31^ | 82 | □ | ■ | □ & ■ | 4·3 | 18 | High-risk, bled at presentation |
| Wang *et al.* 2003^32^ | 137 | □ | ■ | □ | 4·3 | 11 | Brainstem CCM |
| Mathiesen *et al.* 2003^33^ | 68 | □ | ? | □ & ■ | 4·6 | ? | Brainstem CCM |
| Aiba *et al.* 1995^34^ | 110 | □ | ■ | □ | 4·7 | ? | None |
| Li *et al.* 2021^35^ | 708 | □ | ■ | ■ | 4·8 | 12·7 | Brainstem CCM |
| Al-Shahi Salman *et al.* 2012^36^ | 134 | ■ | ■ | ■ | 5·0 | 5·0 | None |
| Moriarity *et al.* 1999^37^ | 68 | □ | ■ | ■ | 5·2 | ? | None |
| Sandmann *et al.* 2024^38^ | 265 | □ | ■ | □ | 6·4 | 22·4 | Conservative management |
| Li *et al.* 2014^39^ | 331 | □ | ■ | ■ | 6·5 | 28·6 | Brainstem CCM |
| Velz *et al.* 2018^40^ | 406 | □ | ■ | □ | 6·8 | 35·0 | Multiple CCMs |
| Flemming *et al.* 2012^41^ | 292 | □ | ■ | □ | 7·0 | 25 | None |
| Flemming *et al.* 2023^42^ | 405 | □ | ■ | □ & ■ | 7·9 | 38·1 | None |
| Flemming *et al.* 2024^43^ | 315 | □ | ■ | □ & ■ | 8·5 | 37·4 | Conservative management |
| Alalfi *et al.* 2023^44^ | 75 | □ | ■ | □ & ■ | 9·9 | 38·1 | Familial CCM |
| Santos *et al.* 2023^45^ | 85 | □ | ■ | □ & ■ | 10·0 | 10·0 | None |
| Galvão *et al.* 2024^46^ | 47 | □ | ■ | □ | 10·5 | ? | Familial CCM |
| Moore *et al.* 2014^47^ | 107 | □ | ? | □ | 12·3 | 25 | Incidentally diagnosed |

CCM, cerebral cavernous malformation; m, months; y, years.

^1^Median

**References**

1. Kim DS, Park YG, Choi JU, Chung SS, Lee KC. An analysis of the natural history of cavernous malformations. *Surg Neurol* 1997; **48**(1): 9–17.

2. Ma L, Zhang S, Li Z, et al. Morbidity After Symptomatic Hemorrhage of Cerebral Cavernous Malformation: A Nomogram Approach to Risk Assessment. *Stroke* 2020; **51**(10): 2997–3006.

3. Labauge P, Brunereau L, Laberge S, Houtteville JP. Prospective follow-up of 33 asymptomatic patients with familial cerebral cavernous malformations. *Neurology* 2001; **57**(10): 1825–8.

4. Carrión-Penagos J, Zeineddine HA, Polster SP, et al. Subclinical imaging changes in cerebral cavernous angiomas during prospective surveillance. *J Neurosurg* 2021; **134**(3): 1147–54.

5. Robinson JR, Awad IA, Little JR. Natural history of the cavernous angioma. *J Neurosurg* 1991; **75**(5): 709–14.

6. Fritschi JA, Reulen HJ, Spetzler RF, Zabramski JM. Cavernous malformations of the brain stem. A review of 139 cases. *Acta Neurochir (Wien)* 1994; **130**(1–4): 35–46.

7. Zabramski JM, Wascher TM, Spetzler RF, et al. The natural history of familial cavernous malformations: results of an ongoing study. *J Neurosurg* 1994; **80**(3): 422–32.

8. Gomez-Paz S, Maragkos GA, Salem MM, et al. Symptomatic Hemorrhage From Cerebral Cavernous Malformations: Evidence from a Cohort Study. *World Neurosurg* 2020; **135**: e477–e87.

9. Kalani MY, Zabramski JM. Risk for symptomatic hemorrhage of cerebral cavernous malformations during pregnancy. *J Neurosurg* 2013; **118**(1): 50–5.

10. Marques LL, Jaeggi C, Branca M, Raabe A, Bervini D, Goldberg J. Bleeding Risk of Cerebral Cavernous Malformations in Patients on Statin and Antiplatelet Medication: A Cohort Study. *Neurosurgery* 2023; **93**(3): 699–705.

11. Ghannane H, Khalil T, Sakka L, Chazal J. [Analysis of a series of cavernomas of the central nervous system: 39 non operated cases, 39 operated cases, 1 dead]. *Neurochirurgie* 2007; **53**(2–3 Pt 2): 217–22.

12. Santos AN, Rauschenbach L, Saban D, et al. Medication intake and hemorrhage risk in patients with familial cerebral cavernous malformations. *J Neurosurg* 2022; **137**(4): 1088–94.

13. Santos AN, Rauschenbach L, Saban D, et al. Multiple cerebral cavernous malformations: Clinical course of confirmed, assumed and non-familial disease. *Eur J Neurol* 2022; **29**(5): 1427–34.

14. Kondziolka D, Lunsford LD, Kestle JR. The natural history of cerebral cavernous malformations. *J Neurosurg* 1995; **83**(5): 820–4.

15. Porter RW, Detwiler PW, Spetzler RF, et al. Cavernous malformations of the brainstem: experience with 100 patients. *J Neurosurg* 1999; **90**(1): 50–8.

16. Jeon JS, Kim JE, Chung YS, et al. A risk factor analysis of prospective symptomatic haemorrhage in adult patients with cerebral cavernous malformation. *J Neurol Neurosurg Psychiatry* 2014; **85**(12): 1366–70.

17. Bervini D, Jaeggi C, Mordasini P, Schucht P, Raabe A. Antithrombotic medication and bleeding risk in patients with cerebral cavernous malformations: a cohort study. *J Neurosurg* 2019; **130**(6): 1922–30.

18. Santos AN, Rauschenbach L, Gull HH, et al. Central nervous system cavernous malformations: cross-sectional study assessing rebleeding risk after a second haemorrhage. *Eur J Neurol* 2023; **30**(1): 144–9.

19. Chen B, Herten A, Saban D, et al. Hemorrhage from cerebral cavernous malformations: The role of associated developmental venous anomalies. *Neurology* 2020; **95**(1): e89–e96.

20. Arauz A, Patiño-Rodriguez HM, Chavarria-Medina M, Becerril M, Longo GM, Nathal E. Rebleeding and Outcome in Patients with Symptomatic Brain Stem Cavernomas. *Cerebrovasc Dis* 2017; **43**(5–6): 283–9.

21. Zuurbier SM, Santos AN, Flemming KD, et al. Female Hormone Therapy and Risk of Intracranial Hemorrhage From Cerebral Cavernous Malformations: A Multicenter Observational Cohort Study. *Neurology* 2023; **100**(16): e1673–e9.

22. Al-Holou WN, O'Lynnger TM, Pandey AS, et al. Natural history and imaging prevalence of cavernous malformations in children and young adults. *J Neurosurg Pediatr* 2012; **9**(2): 198–205.

23. Weinsheimer S, Nelson J, Abla AA, et al. Intracranial Hemorrhage Rate and Lesion Burden in Patients With Familial Cerebral Cavernous Malformation. *J Am Heart Assoc* 2023; **12**(3): e027572.

24. Dammann P, Jabbarli R, Wittek P, et al. Solitary Sporadic Cerebral Cavernous Malformations: Risk Factors of First or Recurrent Symptomatic Hemorrhage and Associated Functional Impairment. *World Neurosurg* 2016; **91**: 73–80.

25. Tian KB, Zheng JJ, Ma JP, et al. Clinical course of untreated thalamic cavernous malformations: hemorrhage risk and neurological outcomes. *J Neurosurg* 2017; **127**(3): 480–91.

26. Goldberg J, Jaeggi C, Schoeni D, Mordasini P, Raabe A, Bervini D. Bleeding risk of cerebral cavernous malformations in patients on β-blocker medication: a cohort study. *J Neurosurg* 2018: 1–6.

27. Porter PJ, Willinsky RA, Harper W, Wallace MC. Cerebral cavernous malformations: natural history and prognosis after clinical deterioration with or without hemorrhage. *J Neurosurg* 1997; **87**(2): 190–7.

28. Barker FG, 2nd, Amin-Hanjani S, Butler WE, et al. Temporal clustering of hemorrhages from untreated cavernous malformations of the central nervous system. *Neurosurgery* 2001; **49**(1): 15–24.

29. Schneble HM, Soumare A, Hervé D, et al. Antithrombotic therapy and bleeding risk in a prospective cohort study of patients with cerebral cavernous malformations. *Stroke* 2012; **43**(12): 3196–9.

30. Kearns KN, Chen CJ, Yagmurlu K, et al. Hemorrhage Risk of Untreated Isolated Cerebral Cavernous Malformations. *World Neurosurg* 2019; **131**: e557–e61.

31. Hasegawa T, McInerney J, Kondziolka D, Lee JY, Flickinger JC, Lunsford LD. Long-term results after stereotactic radiosurgery for patients with cavernous malformations. *Neurosurgery* 2002; **50**(6): 1190–7.

32. Wang CC, Liu A, Zhang JT, Sun B, Zhao YL. Surgical management of brain-stem cavernous malformations: report of 137 cases. *Surg Neurol* 2003; **59**(6): 444–54.

33. Mathiesen T, Edner G, Kihlström L. Deep and brainstem cavernomas: a consecutive 8-year series. *J Neurosurg* 2003; **99**(1): 31–7.

34. Aiba T, Tanaka R, Koike T, Kameyama S, Takeda N, Komata T. Natural history of intracranial cavernous malformations. *J Neurosurg* 1995; **83**(1): 56–9.

35. Li D, Wu ZY, Liu PP, et al. Natural history of brainstem cavernous malformations: prospective hemorrhage rate and adverse factors in a consecutive prospective cohort. *J Neurosurg* 2021; **134**(3): 917–28.

36. Al-Shahi Salman R, Hall JM, Horne MA, et al. Untreated clinical course of cerebral cavernous malformations: a prospective, population-based cohort study. *Lancet Neurol* 2012; **11**(3): 217–24.

37. Moriarity JL, Wetzel M, Clatterbuck RE, et al. The natural history of cavernous malformations: a prospective study of 68 patients. *Neurosurgery* 1999; **44**(6): 1166–71.

38. Sandmann ACA, Kempeneers MA, van den Berg R, Verbaan D, Vandertop WP, Coutinho JM. Clinical course of patients with conservatively managed cerebral cavernous malformations. *Eur Stroke J* 2024; **9**(3): 667–75.

39. Li D, Hao SY, Jia GJ, Wu Z, Zhang LW, Zhang JT. Hemorrhage risks and functional outcomes of untreated brainstem cavernous malformations. *J Neurosurg* 2014; **121**(1): 32–41.

40. Velz J, Stienen MN, Neidert MC, Yang Y, Regli L, Bozinov O. Routinely Performed Serial Follow-Up Imaging in Asymptomatic Patients With Multiple Cerebral Cavernous Malformations Has No Influence on Surgical Decision Making. *Front Neurol* 2018; **9**: 848.

41. Flemming KD, Link MJ, Christianson TJ, Brown RD, Jr. Prospective hemorrhage risk of intracerebral cavernous malformations. *Neurology* 2012; **78**(9): 632–6.

42. Flemming KD, Lanzino G. Are there differences in clinical presentation, radiologic findings, and outcomes in female patients with cavernous malformation? *Acta Neurochir (Wien)* 2023; **165**(7): 1855–61.

43. Flemming KD, Brown RD, Lanzino G. Contemporary cohort of cerebral cavernous malformations: natural history and utility of follow-up MRI. *J Neurosurg* 2024; **141**(5): 1159–67.

44. Alalfi MO, Lanzino G, Flemming KD. Clinical presentation, hemorrhage risk, and outcome in patients with familial cavernous malformations: a pragmatic prospective analysis of 75 patients. *J Neurosurg* 2023; **139**(4): 1018–24.

45. Santos AN, Rauschenbach L, Gull HH, et al. Natural course of cerebral and spinal cavernous malformations: a complete ten-year follow-up study. *Sci Rep* 2023; **13**(1): 15490.

46. Galvão GDF, Neumann VB, Verly G, et al. Clinical features, hemorrhage risk and epilepsy outcomes of familial cerebral cavernous malformation: A 20-year observational pragmatic single-center study. *J Stroke Cerebrovasc Dis* 2024; **33**(12): 108041.

47. Moore SA, Brown RD, Jr., Christianson TJ, Flemming KD. Long-term natural history of incidentally discovered cavernous malformations in a single-center cohort. *J Neurosurg* 2014; **120**(5): 1188–92.
